# Supplementary material for: Air pollution, service development and innovation: Evidence from China
Source: PLoS One. 2022 May 24;17(5):e0263883. doi: 10.1371/journal.pone.0263883 (PMC9129014; doi:10.1371/journal.pone.0263883)
Supplement: Attachment — Submitted filename: Response to Reviewers.docx [file pone.0263883.s002.docx]

Thank the editor and referees for the professional comments. It encourages me again. I feel sorry for my carelessness as subscript and so on. In this version, I revise my manuscript according to the comments. The details are as follows.

## Responses to academic editor

Edit the paper for English language by a native English speaker.

Also revise your introduction and other sections carefully.

Thanks for the editor’s advice. I recheck the paper and then find a native English speaker to improve the expression, especially in the Abstract and Introduction.

I also uploaded my figure in the Preflight Analysis and Conversion Engine (PACE) digital diagnostic.

To satisfy the journal requirements, I rechecked and corrected the reference.

In Line 486 of “Revised Manuscript with Track Changes”.

## Responses to referee 1

Thanks a lot to the authors for taken on board the comments and improving the paper.

I see in some paragraphs 'pm2.5'. It should be corrected to PM2.5 (2.5 in subscript).

Sorry for my carelessness. I subscribe the size of PM2.5 in Lines 31 and 32. There is some other ‘pm25’ for the variable is named ‘pm25’. Thanks again for referee’s careful examination.

## Responses to referee 2

Thanks for the referee’s professional comments, which not only improve the quality of the article, but also provide a great inspiration for our future research.

- Grammar check the title.

I checked the grammar of the title and corrected in the manuscript.

- The first sentence of the introduction is not clear.

I modified the Introduction in the manuscript.

- Lines 31 and 32 subscribe the size of the PM.

I subscribed the size of PM2.5 in Lines 31 and 32 in the new version and I also reexamined the article. I also feel sorry for my carelessness.

- Explain all the abbreviations in its first mention in the text.

I checked the text and finded the expression of "vote with their feet" in Line 64 and "resource trap" in Line 203 may be not clear. Line 64 and Line 203 are in the “Revised Manuscript with Track Changes”. I modified it.

- Revised the journal guidelines to the make sure the manuscript follows the guidelines.

Thanks to the referee’s advice.
